# The estrogen and c-Myc target gene *HSPC111 *is over-expressed in breast cancer and associated with poor patient outcome

**DOI:** 10.1186/bcr1985

**Published:** 2008-03-29

**Authors:** Alison J Butt, C Marcelo Sergio, Claire K Inman, Luke R Anderson, Catriona M McNeil, Amanda J Russell, Marco Nousch, Thomas Preiss, Andrew V Biankin, Robert L Sutherland, Elizabeth A Musgrove

**Affiliations:** 1 Cancer Research Program, Garvan Institute of Medical Research, St. Vincent's Hospital, Victoria Street, Darlinghurst, New South Wales 2010, Australia; 2 St. Vincent's Clinical School, Faculty of Medicine, University of New South Wales, Sydney, New South Wales 2052 Australia; 3 Molecular Genetics Program, Victor Chang Cardiac Research Institute, Victoria Street, Darlinghurst, New South Wales 2010, Australia

## Abstract

**Introduction:**

Estrogens play a pivotal role in the initiation and progression of breast cancer. The genes that mediate these processes are not fully defined, but potentially include the known mammary oncogene *MYC*. Characterization of estrogen-target genes may help to elucidate further the mechanisms of estrogen-induced mitogenesis and endocrine resistance.

**Methods:**

We used a transcript profiling approach to identify targets of estrogen and c-Myc in breast cancer cells. One previously uncharacterized gene, namely HBV pre-S2 trans-regulated protein 3 (*HSPC111*), was acutely upregulated after estrogen treatment or inducible expression of c-Myc, and was selected for further functional analysis using over-expression and knock-down strategies. HSPC111 expression was also analyzed in relation to *MYC *expression and outcome in primary breast carcinomas and published gene expression datasets.

**Results:**

Pretreatment of cells with c-Myc small interfering RNA abrogated estrogen induction of *HSPC111*, identifying *HSPC111 *as a potential c-Myc target gene. This was confirmed by the demonstration of two functional E-box motifs upstream of the transcription start site. HSPC111 mRNA and protein were over-expressed in breast cancer cell lines and primary breast carcinomas, and this was positively correlated with *MYC *mRNA levels. HSPC111 is present in a large, RNA-dependent nucleolar complex, suggesting a possible role in ribosomal biosynthesis. Neither over-expression or small interfering RNA knock-down of HSPC111 affected cell proliferation rates or sensitivity to estrogen/antiestrogen treatment. However, high expression of *HSPC111 *mRNA was associated with adverse patient outcome in published gene expression datasets.

**Conclusion:**

These data identify *HSPC111 *as an estrogen and c-Myc target gene that is over-expressed in breast cancer and is associated with an adverse patient outcome.

## Introduction

Breast cancer is the major contributor to cancer incidence and mortality in women in the Western world. Although the genetic and environmental factors that lead to the initiation of breast cancer remain unclear, it is known that exposure to estrogens plays a crucial role in the development and progression of this disease [1]. It has been proposed that the causative link between estrogen and breast cancer is due to its potent mitogenic and antiapoptotic effects [2]. However, it is not fully understood how these effects are mediated at the molecular level. Such insight may provide clues to the mechanisms of estrogen-induced mitogenesis and cell survival, or resistance to endocrine therapies, or identify potential novel therapeutic targets for breast cancer, particularly in the settings of endocrine insensitivity and resistance. Thus, the identification and characterization of estrogen target genes is a major research priority.

The majority of breast cancers (about 75%) are estrogen receptor (ER)-positive, and estrogen is a potent mitogen for human breast cancer cells *in vitro*. The proliferation of ER-positive MCF-7 breast cancer cells in culture is inhibited by antiestrogens, and this effect is reversed by estrogen. Estrogen and antiestrogens regulate cell cycle entry and rates of progression during early G_1 _phase [3-5], and this is effected by modulation of G_1 _cyclin gene expression and activation of cyclin-dependent kinases 2 and 4 [6,7]. In addition, there is now evidence of converging activation of downstream estrogen signaling through crosstalk with growth factor-activated tyrosine kinase receptors [8]. Thus, there are compelling data suggesting that estrogen can mediate its growth effects by influencing the expression and function of genes critical to cell proliferation, by both 'genomic' and 'nongenomic' (cytoplasmic signaling) mechanisms [9].

One of the earliest transcriptional responses to estrogen is increased *MYC *expression [10]. Myc is a nuclear transcription factor that exhibits high-affinity and site-specific DNA-binding activity when complexed with its cellular partner Max, and it is rate-limiting for cell cycle progression through G_1 _phase [11], mediated in part through its effects on activation of cyclin-dependent kinases [12,13]. Inhibition of c-Myc expression abrogates estrogen-stimulated breast cancer cell proliferation [14], and blocks cell cycle progression leading to a G_1 _arrest [15]. Estrogen-regulated induction of *MYC *may play a critical role in the initiation of breast tumorigenesis, because *MYC *was the first mammary oncogene to be demonstrated by transgenesis [16]. These data strongly implicate c-Myc as an important mediator of the mitogenic function of estrogen, with a potential role in the initiation and progression of breast cancer. This concept is supported by studies demonstrating that Myc over-expression confers resistance to antiestrogens *in vitro *[17,18], and that inducible expression of c-Myc can replace estrogen in reinitiating cell cycle progression in antiestrogen-arrested breast cancer cells [12].

Because c-Myc can mimic the effects of estrogen on cell cycle progression in MCF-7 cells [12], we examined the transcriptional response to estrogen and to inducible c-Myc to identify novel targets of both estrogen and c-Myc in breast cancer cells (Musgrove EA, Sergio CM, Butt AJ, Sutherland RL; unpublished data). Here, we report an initial characterization of one such gene, namely HBV pre-S2 trans-regulated protein 3 (*HSPC111*). These studies reveal that *HSPC111 *is a direct transcriptional target of Myc, which is localized in the nucleolus and is over-expressed in several common cancers. Furthermore, elevated expression of HSPC111 is associated with reduced survival in breast cancer patients.

## Materials and methods

### Breast cancer cell lines and tissue samples

The human breast cancer cell line, MCF-7, was routinely maintained in RPMI-1640 medium supplemented with 10% fetal calf serum, 10 μg/ml insulin and 2.92 mg/ml glutamine under standard conditions. The human breast cancer mRNA samples utilized in this study have previously been described [19].

### Quantitative real-time PCR

Total RNA was isolated using the RNAeasy kit (Qiagen Pty Ltd, Victoria, Australia) from cells pretreated with ICI 182780 (7α-[9-(4,4,5,5,5-pentafluoropentylsulfinyl) nonyl] estra-1,3,5,(10)-triene-3,17β-diol), which was a kind gift from Dr Alan Wakeling (Astra-Zeneca Pharmaceuticals, Alderly Park, Cheshire, UK), and then treated with 17β-estradiol, zinc, or vehicle. Quantitative real-time PCR was performed using the ABI Prism 7900HT Sequence Detection System (Applied Biosystems, Foster City, CA, USA) using Taq-Man^® ^probes for *HSPC111 *(Applied Biosytems). Data analyses were performed using the ΔCt method with *RPLP0 *(Applied Biosystems) as internal loading control. Fold changes in gene expression were calculated relative to the 0 hours time point. For correlation experiments, total RNA from a panel of breast cancer cell lines was isolated and quantitative real-time PCR was performed using Taq-Man^® ^probes for *MYC *and *HSPC111*. Correlation was performed using standard linear regression analysis.

### Immunoblot analysis

Cell lysates were collected as described previously [6]. Antibodies used were HSPC111 (see below) or V5 (Invitrogen Life Technologies Inc., Carlsbad, CA, USA). Glyceraldehyde 3-phosphate dehydrogenase (GAPDH; Ambion, Austin, TX) or actin (Sigma, St Louis, MO, USA) was used as loading control.

### Constructs

The sequence between -799 and +43 base pairs (bp) of the *HSPC111 *promoter was amplified by nested PCR from MCF-7-derived genomic DNA. The resulting 842 bp fragment was cloned into pGL3-Basic reporter construct (Promega, Madison, WI, USA).

### Luciferase reporter assays

MCF-7 cells were transfected using Lipofectamine 2000 (Invitrogen) with luciferase reporter construct, renilla luciferase reporter construct, pRLSV40 (Promega), and either the c-Myc expression plasmid pCDNA3.1-cMyc or pcDNA3.1. Transfected cells were stimulated with increasing concentrations of zinc (up to 80 μmol/l) for 6 hours before harvesting. Luciferase activity was assayed 24 hours after transfection using the Dual-Luciferase Reporter Assay System (Promega) and normalized to renilla luciferase activity. All values are relative to the activity of the pGL3-Basic reporter.

### Electrophoretic mobility shift assays (EMSAs)

The sequences of the oligonucleotides used to investigate the three putative c-Myc binding sites in the *HSCP111 *promoter were as follows: HSPCsite1(TOP): 5'-CTAGGAGGCCCATGTGTCGCTG-3' ; HSPCsite1(BOT): 5'-CTAGCAGCGACACATGGGCCTC-3' ; HSPCsite2(TOP): 5'-CTAGGGCTCACACCTGTAATCC-3' ; HSPCsite2(BOT): 5'-CTAGGGATTACAGGTGTGAGCC-3' ; HSPCsite3(TOP): 5'-CTAGGCGGATCACCTGAGGTCA-3' ; HSPCsite3(BOT): 5'-CTAGTGACCTCAGGTGATCCGC-3' ; CAD(TOP): 5'-CTAGGTTAGCCACGTGGACCGA-3' ; and CAD(BOT): 5'-CTAGTCGGTCCACGTGGCTAAC-3'. The annealed oligonucleotides were radiolabeled with [α-^32^P]dCTP using Klenow fragment. Electrophoretic mobility shift assays were performed using nuclear extracts from MCF-7 cells. Equal amounts of nuclear extracts were incubated with the radiolabeled oligonucleotides following standard protocols, resolved on a 5% acrylamide gel and visualized by autoradiography. Competition assays were performed using 100-fold excess of competitor unradiolabeled oligonucleotides. The following oligonucleotides were used as nonspecific competitor oligonucleotides: 5'-CTAGTCTACTCCACTGCTGTCTATC-3' and 5'-CTAGGATAGACAGCAGTGGAGTAGA-3'.

### Chromatin immunoprecipitation assays

Chromatin immunoprecipitation (ChIP) assays were performed on chromatin from MCF-7/MycWT cells using a ChIP Assay Kit (Upstate Biotechnology, Millipore Corp. Billerica, MA, USA), following the manufacturer's instructions. Complexes were immunoprecipitated with c-Myc antibodies (9E10, C-33; Santa Cruz Biotechnology, Santa Cruz, CA, USA) or a nonspecific PICK-1 antibody (Santa Cruz Biotechnology). The oligonucleotides used to detect the putative c-Myc binding sites in the HSPC111 promoter were as follows: HSPC111-ChIP P1: 5'-GAGTTTATTAAGCAGGGGAGTGGAG-3' ; HSPC111-CHIP P2: 5'-CCGCAGAAATGATTCCAAAACC-3', for site 1; HSPC111-CHIP P3: 5'-GTTGGTCAGGCTGGTCTTGAAC-3' ; HSPC111-ChIP P4: 5'-CGGACTTTGGAGTGGTGCTTAG-3', for site 3. For the analysis using quantitative real-time PCR, the following oligonucleotides were used: HSPC111-QPCR P1: 5'-TCCGCAGAAATGATTCCAAAA-3' ; and HSPC111-QPCR: P2 5'-AAGGGTCACTTCCTCCCCAG-3'.

### Stable transfection

Full-length HSPC111 cDNA was generated by reverse transcription PCR from MCF-7 cells, and cloned into pDONR221 (Invitrogen). Constructs were recombined with the Gateway destination vector pcDNA3.1/nV5-pDEST (amino-terminal V5 fusion; Invitrogen), and then transfected into MCF-7 using Fugene-6 transfection reagent (Roche Applied Science, Indianapolis, IN, USA). Clones (MCF/HSPC) were selected and expanded in the presence of Geneticin (800 μg/ml; Invitrogen). Cells transfected with a pcDNA3.1/nV5-pDEST-LacZ vector were used as control (MCF/LacZ). MCF-7 cells inducibly expressing c-Myc wild-type (MCF/MycWT) or empty vector control cells were generated as previously described [12].

### HSPC111-specific antibody production

Amino-terminal HIS-tagged HSPC111 was expressed in *Escherichia coli *BL21 (DE3) pLysS. Cultures were lyzed and proteins elutions were pooled for polyclonal antibody production in rabbits. Initial bleeds were purified using a protein A column and optimized for immunoblot analysis. Antibody specificity was confirmed in MCF/HSPC-NV5 cells by comparison with V5-tagged protein detected by immunocytochemisty and immunoblotting. The antibody detected both endogenous and V5-tagged HSPC-111 protein.

### Cell proliferation and S phase analysis

HSPC111-expressing cells and LacZ controls were plated at 1 × 10^5 ^(day 0) and subsequently harvested and counted up to day 5. Exponentially growing MCF-7 cells expressing HSPC111 or LacZ controls were treated with 1 μmol/l 4-hydroxytamoxifen (Sigma), 10 nmol/l ICI 182780 or vehicle (ethanol) for 48 hours. Cells were harvested and S phase was analyzed by propidium iodide staining and flow cytometry.

### Small interfering RNA

Small interfering (si)RNAs (siMyc17: D-003282-17-0050; siHSPC2: D-016096-02-0050; siHSPC4: D-016096-04-0050; siCONTROL RISC-Free siRNA: D-001220-01-20; and siRNA nontargeting control 2: D-001210-02-20) were purchased from Dharmacon (Lafayette, CO, USA) and transfected using Lipofectamine 2000 (Invitrogen). For estrogen 'rescue' experiments, cells were pretreated with ICI 182780 (10 nmol/l) at 24 hours after transfection and 48 hours later were treated with vehicle (ethanol) or 17β estradiol (100 nmol/l).

### Immunofluorescence

Parental MCF-7 cells or those expressing V5-tagged HSPC111 were stained with anti-HSPC111, anti-V5 (Invitrogen), anti-nucleophosmin or anti-fibrillarin (Santa Cruz Biotechnology) antibodies and DAPI (4,6-diamidino-2-phenylindole), and were visualized using confocal microscopy.

### Sucrose density gradient fractionation

Nuclear extracts from exponentially growing MCF-7 cells were separated by sucrose density gradient fractionation as described previously [20]. The gradients were analyzed through a UV monitor for continuous measurement of the absorbance at 254 nm and fractions collected. For immunoblot analysis, proteins from each fraction were precipitated with cold trichloroacetic acid at a final concentration of 10%.

### Survival analyses

Datasets from two breast cancer cohorts using two different methodologies to analyze global gene expression were accessed. The first (referred to as the Uppsala cohort) is a group of 236 breast cancer patients [21] whose tumor RNA was analyzed using Affymetrix Genechip^® ^(Affymetrix Inc., Santa Clara, CA, USA) HGU133A and B microarrays (NCBI GEO accession GSE3494; files were GSE3494-GPL96_series_matrix.txt.gz [HG U133A] and GSE3494-GPL97_series_matrix.txt.gz [HGU133B]). The second (from The Nederlands Kanker Instituut and designated the NKI cohort [22]) contained 295 cases that were assessed using Rosetta NKI spotted oligonucleotide arrays [23]. Datasets from both published series had complete data for clinicopathological variables and ER, progesterone receptor and HER2/neu status, as well as disease-specific survival. Univariate and multivariate analyses were performed as previously described [24] to assess the association of *HSPC111 *and *MYC *expression with survival using Statview 5.0 Software (Abacus Systems, Berkeley, CA, USA). *P *< 0.05 was considered statistically significant. The outcome variables were assessed as time to event, which was defined as the difference between the time of diagnosis and the time of death from breast cancer. Kaplan-Meier analysis was used for univariate analysis and to plot survival curves. Cox proportional hazards models were used to estimate hazard ratio (and its 95% confidence interval [CI]) associated with each risk factor and covariate and were also used for multivariate analyses.

## Results

### Identification of HSPC111 as an estrogen-regulated c-Myc target

**Figure 1:**
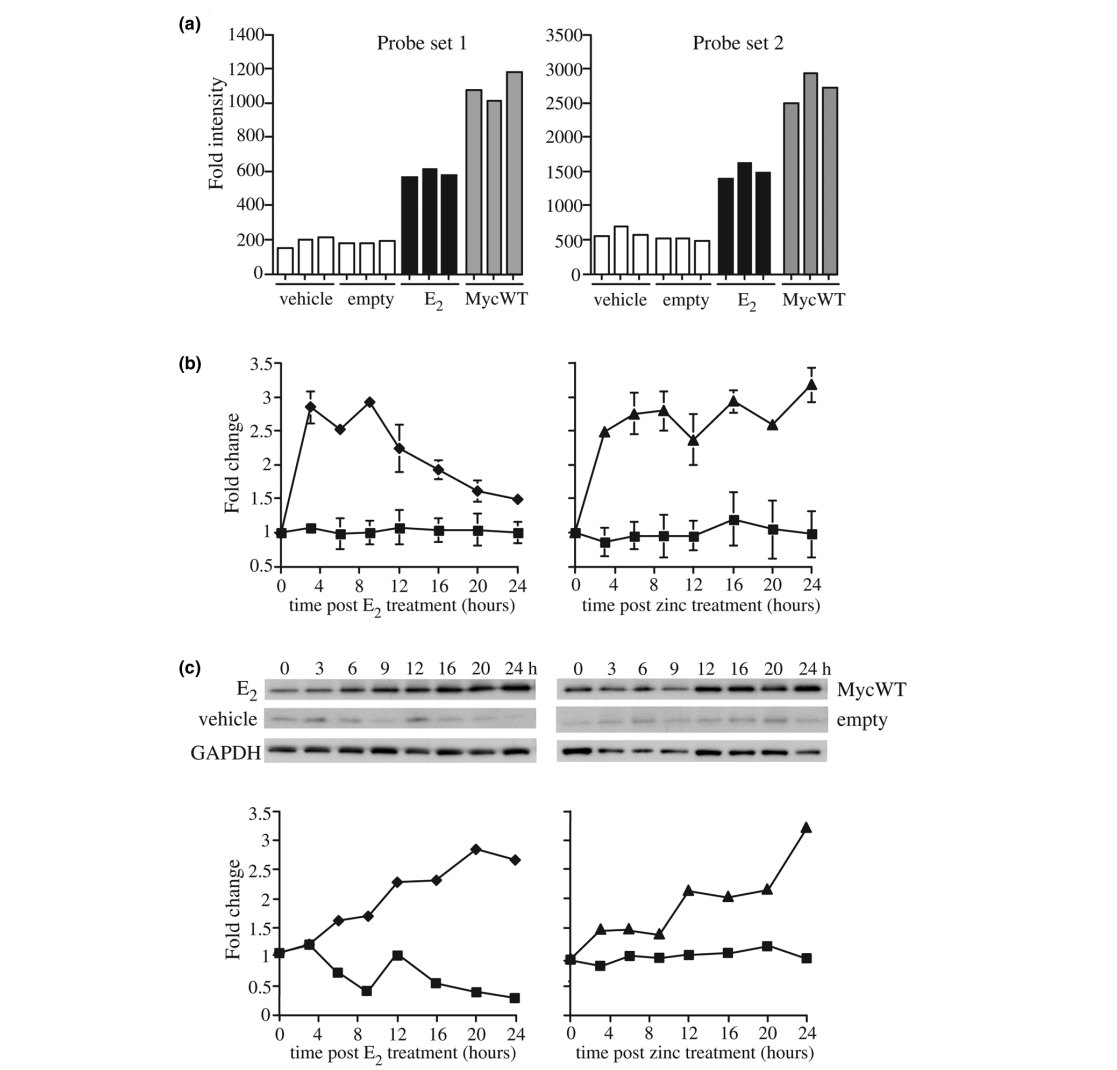
Identification of HSPC111 as an estrogen-regulated target of c-Myc. Cells were pretreated with ICI 182780 for 48 hours. Parental MCF-7 cells were then treated with either 17β-estradiol (diamonds) or vehicle (squares), and MCF-7/MycWT (triangles) and empty vector (squares) cells were treated with zinc. **(a) ***HSPC111 *mRNA expression in two probe sets from HG-U133 Plus V2.0 microarray platforms, 6 hours after treatment with estradiol (E_2_) or vehicle, or after expression of c-Myc (MycWT) or zinc treatment of empty vector cells (empty). **(b) **RNA was isolated at various time points as indicated and analyzed in triplicate by reverse transcription PCR with *HSPC111*-specific primers. Expression of *HSPC111 *is presented normalized to *RPLP0*. **(c) **immunoblot analysis of endogenous HSPC111 expression in whole cell lysates at time points up to 24 hours. Glyceraldehyde 3-phosphate dehydrogenase (GAPDH) was used as a loading control. Representative blots and densitometric analyses from three independent experiments are shown.

We have established an *in vitro *model to identify novel, estrogen-regulated targets of c-Myc in breast cancer cells [12]. RNA was collected from MCF-7 cells 6 hours after treatment with estrogen or after induction of c-Myc, and differential gene expression was determined using Affymetrix GeneChip Arrays (HG-U133 Plus V2.0) and Bayesian linear modeling methods in the *limma *package [25]. Candidate genes were selected from the list of probes that were significantly upregulated by both estrogen and c-Myc. The previously uncharacterized gene *HSPC111 *was among the most highly induced mRNAs identified in this analysis. Figure 1a shows the intensity of two probe sets for *HSPC111 *that were significantly upregulated by both estrogen and c-Myc.

We confirmed the upregulation of HSPC111 by estrogen and c-Myc over a time course of treatment with 17β-estradiol or induction of c-Myc expression. MCF-7 cells were treated with 17β-estradiol or zinc (Myc or empty vector), and HSPC111 mRNA and protein expression was determined by quantitative real-time PCR and immunoblot, respectively. *HSPC111 *mRNA was rapidly induced (within 3 hours) after estrogen treatment or induced c-Myc expression compared with controls (Figure 1b), and reached a maximal 2.5-fold to 3-fold increase. Immunoblot analysis (Figure 1c) showed a similar increase in expression of endogenous HSPC111 protein after treatment with estrogen or induced c-Myc expression.

### Estrogen's effects on HSPC111 expression are dependent upon Myc

**Figure 2:**
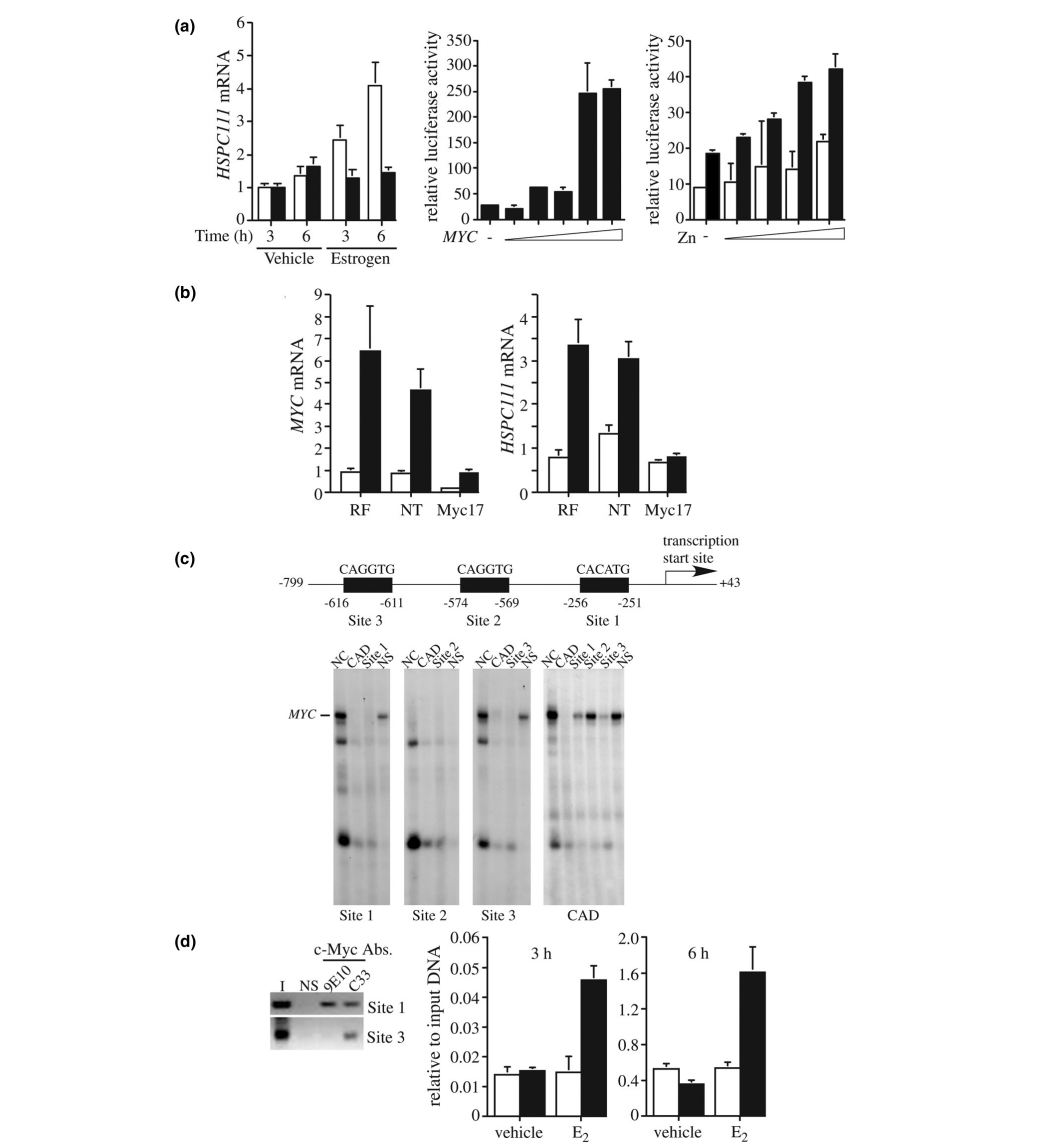
Estrogen regulation of HSPC111 expression is dependent upon direct transcriptional activation by Myc. **(a) **Left panel: MCF-7 cells were arrested with ICI 182780 for 48 hours and then treated with cycloheximide (black bars) or control (white bars) before addition of estrogen or vehicle for 3 and 6 hours; levels of *HSPC111 *mRNA were determined by quantitative real-time PCR. Middle panel: MCF-7 cells were transfected with an *HSPC111*-luciferase reporter construct in the presence of increasing amounts of the c-Myc expression construct pCDNA3.1-cMyc. Right panel: MCF-7/MycWT (black bars) and empty vector controls (white bars) were transfected with the *HSPC111*-luciferase reporter construct and stimulated with increasing concentrations of zinc. Values are expressed as means ± standatrd deviation of triplicate samples from three independent experiments. **(b) **Left panel: MCF-7 cells were transfected with Myc-specific small interfering (si)RNA (siMyc17), RISC-free (RF), or nontargeting (NT) siRNA controls, or mock transfected with no siRNA. Transfected cells were arrested with ICI 182780 for 48 hours. Levels of *MYC *and *HSPC111 *mRNA were determined by quantitative real-time PCR after 24 hours of treatment with estradiol (black bars) or vehicle control (white bars). **(c) **Schematic showing the structure of the *HSPC111 *proximal promoter with the location of putative Myc-binding sites (E-boxes). Electrophoretic mobility shift assays demonstrate specific binding of c-Myc to E-boxes within the *HSPC111 *promoter. Radiolabeled oligonucleotides, as indicated above each gel, were incubated with nuclear extract from MCF-7 cells. Lane NC indicates no competitor oligonucleotides were added. The nonlabeled competitor oligonucleotides are indicated below each lane. Lane NS indictates nonspecific competitor oligonucleotide. **(d) **Chromatin was obtained from MCF-7/MycWT cells after 6 hours of treatment with zinc, and immunoprecipitated with c-Myc-specific or nonspecific (NS) antibodies as indicated. Left panel: Chromatin immunoprecipitation (ChIP) assay demonstrating the binding of c-Myc to the endogenous *HSPC111 *promoter. Lane I contains input chromatin that was not immunoprecipitated. Specific regions were then amplified by PCR using primers specific for site 1 or site 3, as indicated. Right panel: ChIP assay demonstrating the recruitment of c-Myc to the endogenous *HSPC111 *promoter in response to treatment with estradiol (E_2_) at 3 and 6 hours. Chromatin was immunoprecipitated with either a c-Myc-specific (C33; black bars) or a nonspecific (white bars) antibody and analyzed by quantitative real-time PCR using primers specific for site 1.

Initial experiments indicated that estrogen induction of HSPC111 mRNA was dependent on ongoing protein synthesis, because it did not occur in the presence of cycloheximide (Figure 2a). We then considered whether the effects of estrogen on HSPC111 expression are mediated via c-Myc. First, we determined whether c-Myc stimulated transcription from the *HSCP111 *promoter. A luciferase reporter construct containing 800 bp upsteam of the *HSPC111 *transcriptional start site was transfected into MCF-7 cells with increasing amounts of a c-Myc expression vector (Figure 2a). Co-expression of c-Myc resulted in a greater than sevenfold increase in luciferase activity. Increased luciferase expression was also observed in MCF-7/Myc cells upon zinc treatment compared with empty vector controls, particularly at higher concentrations of zinc (Figure 2a).

To determine whether estrogen's upregulation of HSPC111 is mediated via Myc, we examined the effects of estrogen on HSPC111 expression in the presence of Myc siRNA. MCF-7 cells were transfected with Myc-specific (siMyc17), RISC-free (RF), or nontargeting (NT) siRNA, and then arrested with ICI 182780 for 48 hours and stimulated with estradiol for 24 hours, when levels of *MYC *and *HSPC111 *mRNA expression were determined. The stimulation of *MYC *mRNA and protein was attenuated in the presence of siMyc17 (Figure 2b and data not shown). However, although *HSPC111 *expression was elevated in controls, there was no significant estrogen-mediated stimulation of *HSPC111 *mRNA in the siMyc17-treated cells (Figure 2b), indicating that estrogen stimulation of HSPC111 expression is dependent upon Myc expression increasing above the level achieved in the presence of siMyc.

### HSPC111 is a direct transcriptional target of c-Myc

The rapid upregulation of *HSPC111 *mRNA by c-Myc suggested that it may be a direct transcriptional target of Myc. Analysis of the human *HSPC111 *genomic sequence revealed three sequences similar to the E-box consensus (CACATG) upstream of the transcriptional start site (Figure 2c). To determine whether c-Myc was able to bind to any of these putative E-boxes, a series of electrophoretic mobility shift assays were performed using double-stranded, radiolabeled oligonucleotides that encompass each of these sites or a known Myc consensus site from the carbamoyl phosphate synthetase-aspartate transcarbamylase-dihydroorotase (CAD) promoter. A band was identified in extracts incubated with radiolabeled oligonucleotides corresponding to sites 1, 3 and CAD, but not site 2 (Figure 2c). A competition assay with radiolabeled CAD oligonucleotide confirmed this as a Myc-specific band. Site 1 and 3 oligonucleotides were able to compete for c-Myc binding to a greater extent than either site 2 or a nonspecific oligonucleotide.

To establish whether the endogenous *HSPC111 *promoter is bound by c-Myc, ChIP assays were performed. Myc expression was induced in MCF-7/MycWT cells by zinc 6 hours before the ChIP assay being performed. Complexes were immunoprecipitated with c-Myc-specific antibodies raised to different epitopes or a nonspecific antibody, and then amplified by PCR using primers specific for site 1 or site 3. Both sites were successfully amplified from the chromatin that was immunoprecipitated with c-Myc antibodies but not with the nonspecific antibody (Figure 2d), suggesting that sites 1 and 3 in the *HSPC111 *promoter are *bona fide *c-Myc binding sites. We further determined whether stimulation with estrogen results in the recruitment of c-Myc to the endogenous *HSPC111 *promoter. Uninduced MCF-7/MycWT cells were treated with either 17β-estradiol or vehicle for 3 and 6 hours before performance of the ChIP assay with site 1 specific primers. Stimulation of the cells with estrogen resulted in a 4.5-fold enhancement in the binding of c-Myc to the *HSPC111 *promoter (Figure 2d).

### HSPC111 localizes to the nucleolus

**Figure 3:**
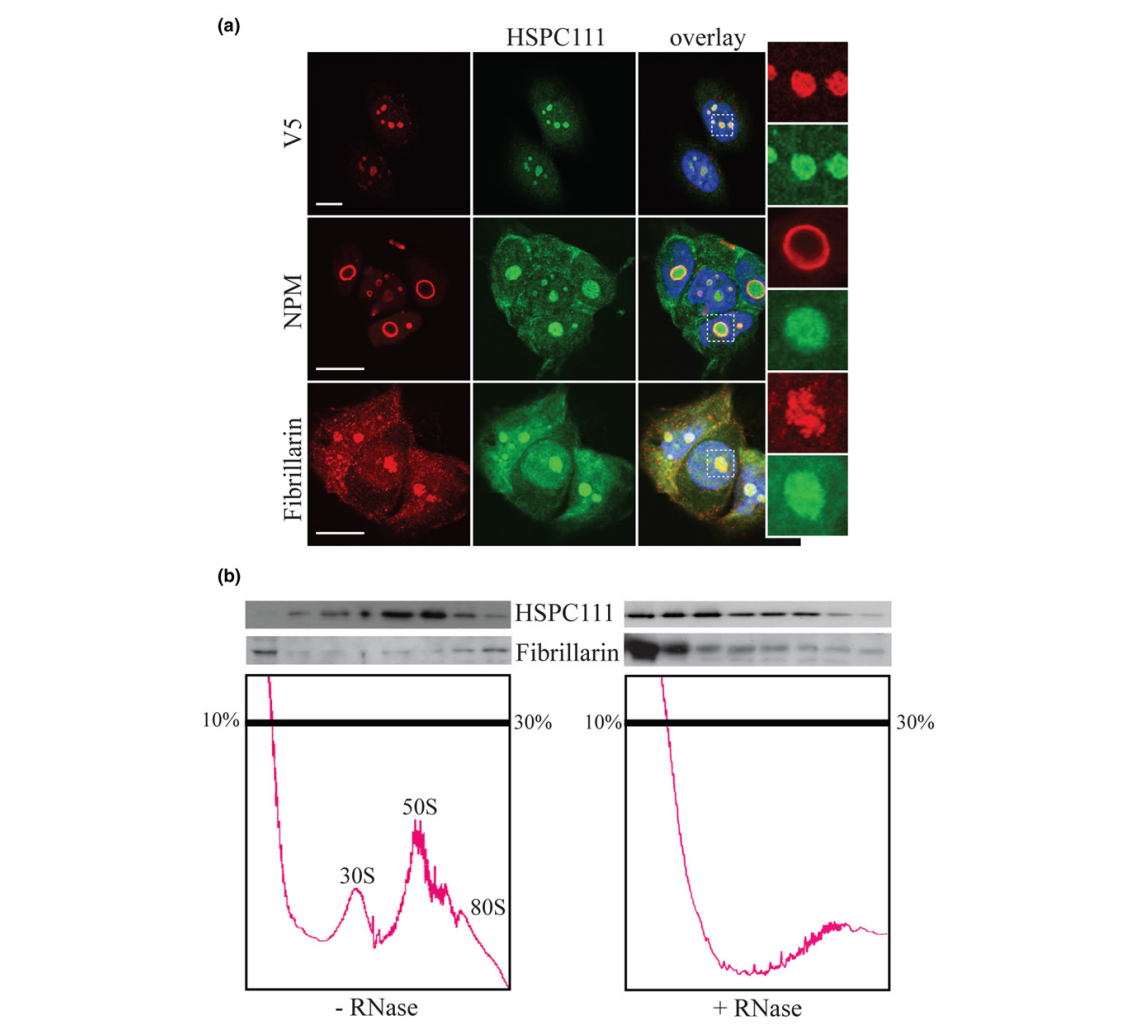
HSPC111 resides in high molecular weight protein complexes in the nucleolus. **(a) **Detection of endogenous and tagged HSPC111 by indirect immunofluorescence. Upper panels: Immunostaining of MCF-7/HSPC-NV5 cells with purified antibodies against endogenous protein (HSPC111; green) and the V5 tag (V5; red). Middle panels: Parental MCF-7 cells were stained with anti-HSPC111 (green) and anti-nucleophosmin (NPM; red) antibodies. Lower panels: Parental MCF-7 cells stained with anti-HSPC111 (green) and anti-fibrillarin (red) antibodies. DNA was counterstained with DAPI (4,6-diamidino-2-phenylindole; blue). Images are representative of at least two independent experiments. Bar = 10 μm. **(b) **Nuclear extracts of MCF-7 cells treated with or without RNase A were fractionated on sucrose density gradients. The trace from continuous monitoring of absorbance at 254 nm is shown. Fractions were precipitated and immunoblotted for HSPC111 and fibrillarin.

Next, we performed a series of experiments aimed at delineating a possible function for HSPC111. HSPC111 has been identified as a nucleolar protein of undefined function [26,27]. We examined this more closely in parental MCF-7 cells and those expressing V5-tagged HSPC111 by indirect immunofluorescence using antibodies against endogenous HSPC111 protein and the V5 tag. Figure 3a demonstrates prominent immunoreactivity of both endogenous and tagged HSPC111 in the nucleolus. Markers of different functional components of the nucleolus were used to define further its subnucleolar localization and hence provide insight into its possible function. The granular region of the nucleolus contains maturing ribosomes and can be identified by immunostaining with nucleophosmin (NPM)/B23, which is an abundant nucleolar phosphoprotein involved in mediating pre-rRNA processing [28]. Figure 3a shows no colocalization of HSPC111 and NPM/B23 within the nucleolus, indicating that HSPC111 is not present in the granular region and that HSPC111 and NPM/B23 probably reside within distinct protein complexes. Antibodies against fibrillarin were then used to label the dense fibrillar components, the site of newly synthesized preribosomal RNA [29]. Dual staining for fibrillarin and HSPC111 expression again demonstrated that each protein resided in distinct compartments of the nucleolus (Figure 3a), which suggests that HSPC111 is not localized in the dense fibrillar component.

### HSPC111 is a component of a large ribonucleoprotein particle

The nucleolus contains many large, multiprotein complexes with a variety of roles, principally in ribosomal biosynthesis. We next addressed whether HSPC111 might be contained within a ribonucleoprotein complex. Nuclear extracts from MCF-7 cells were subjected to sucrose density gradient fractionation to separate various nucleoprotein complexes, and then immunoblotted for HSPC111 expression. A significant proportion of HSPC111 was found in fractions of the gradient that also contained the 30S and 50S pre-preribosomal particles [29]. In contrast, fibrillarin was present in both the low molecular weight fractions at the top of the gradient and at the bottom of the gradient, near the 80S pre-preribosomal particle (Figure 3b). Following treatment with RNaseA to disrupt ribonucleoprotein particles, the sedimentation of both HSPC111 and fibrillarin predominantly shifted toward the top of the gradient (Figure 3b). The loss of pre-ribosomal peaks confirmed the disruption of ribonucleoprotein particles. Together these data indicate that, like fibrillarin, HSPC111 resides in a large multiprotein complex that requires RNA for its integrity.

### Effects of modulation of HSPC111 expression on cell cycle progression

**Figure 4:**
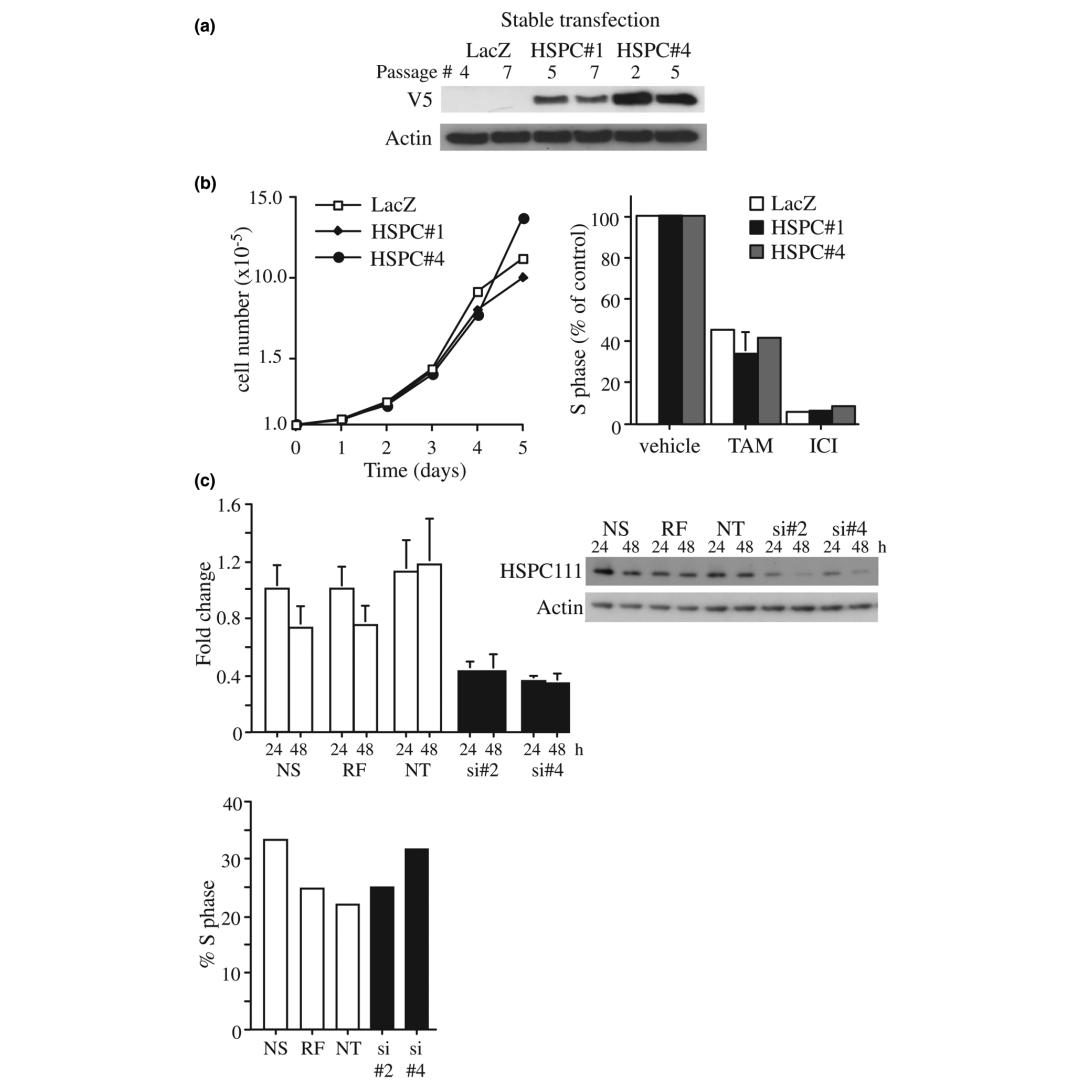
Effects of modulation of HSPC111 expression on cell proliferation. **(a) **Immunoblot analysis of whole cell lysates from MCF-7 clones stably expressing HSPC111 (HSPC#1 and HSPC#4) or LacZ controls at various passages after transfection. Blots were analyzed for expression of tagged HSPC111 protein using V5 antibody or actin as a loading control. **(b) **Left panel: Growth curves of HSPC111 over-expressing clones and LacZ controls. Right panel: Stable transfectants were treated with tamoxifen (TAM), ICI 182780 (ICI), or vehicle for 48 hours, and then S phase was determined by flow cytometric analysis of propidium iodide-stained cells. **(c) **Upper panel: Endogenous HSPC111 mRNA and protein expression in MCF-7 cells 24 and 48 hours after transfection with HSPC111-specific small interfering (si)RNA (siHSPC2 and siHSPC4) determined by quantitative real-time PCR and immunoblot analysis with HSPC111 antibody, respectively. NS indicates mock transfection with no siRNA, RF indicates RISC-free control siRNA, and NT indicates nontargeting control siRNA. Lower panel: S phase was determined 48 hours after transfection by flow cytometric analysis of propidium iodide-stained cells.

Given the proliferative role of Myc in our model and the well characterized link between RNA biosynthesis and cell proliferation [30], we asked whether HSPC111 could recapitulate this aspect of the Myc phenotype. This was examined using two clones of MCF-7 cells constitutively expressing HSPC111 (clones 1 and 4) and LacZ vector alone controls (Figure 4a). Cell proliferation assays exhibited no significant differences in cell number between HSPC111-expressing cells and controls up to 5 days after seeding (Figure 4b). We also examined the effect of HSPC111 expression on antiestrogen-induced cell cycle arrest. HSPC111-expressing cells were treated with tamoxifen or ICI 182780 for 48 hours then cell cycle progression was examined by flow cytometric analysis of propidium iodide stained cells. Treatment of LacZ control cells with antiestrogens resulted in an accumulation of cells in G_1 _phase and a decrease in the percentage of cells in S phase. Constitutive expression of HSPC111 did not significantly alter this response pattern (Figure 4b).

The effect of decreased HSPC111 expression on the proliferation of MCF-7 cells was examined using HSPC111-specific siRNAs. Transfection of MCF-7 cells with two HSPC111-specific siRNAs (siHSPC2 and siHSPC4) resulted in a significant decrease in HSPC111 mRNA and protein expression at 24 and 48 hours compared with controls (Figure 4c). However, inhibition of endogenous HSPC111 expression had no significant effect on cell cycle progression in proliferating cells (Figure 4c). Thus, we concluded that HSPC111 did not have a significant role in mediating Myc-induced proliferation.

### HSPC111 and c-Myc are over-expressed and correlated in human breast cancer cell lines and tissues

**Figure 5:**
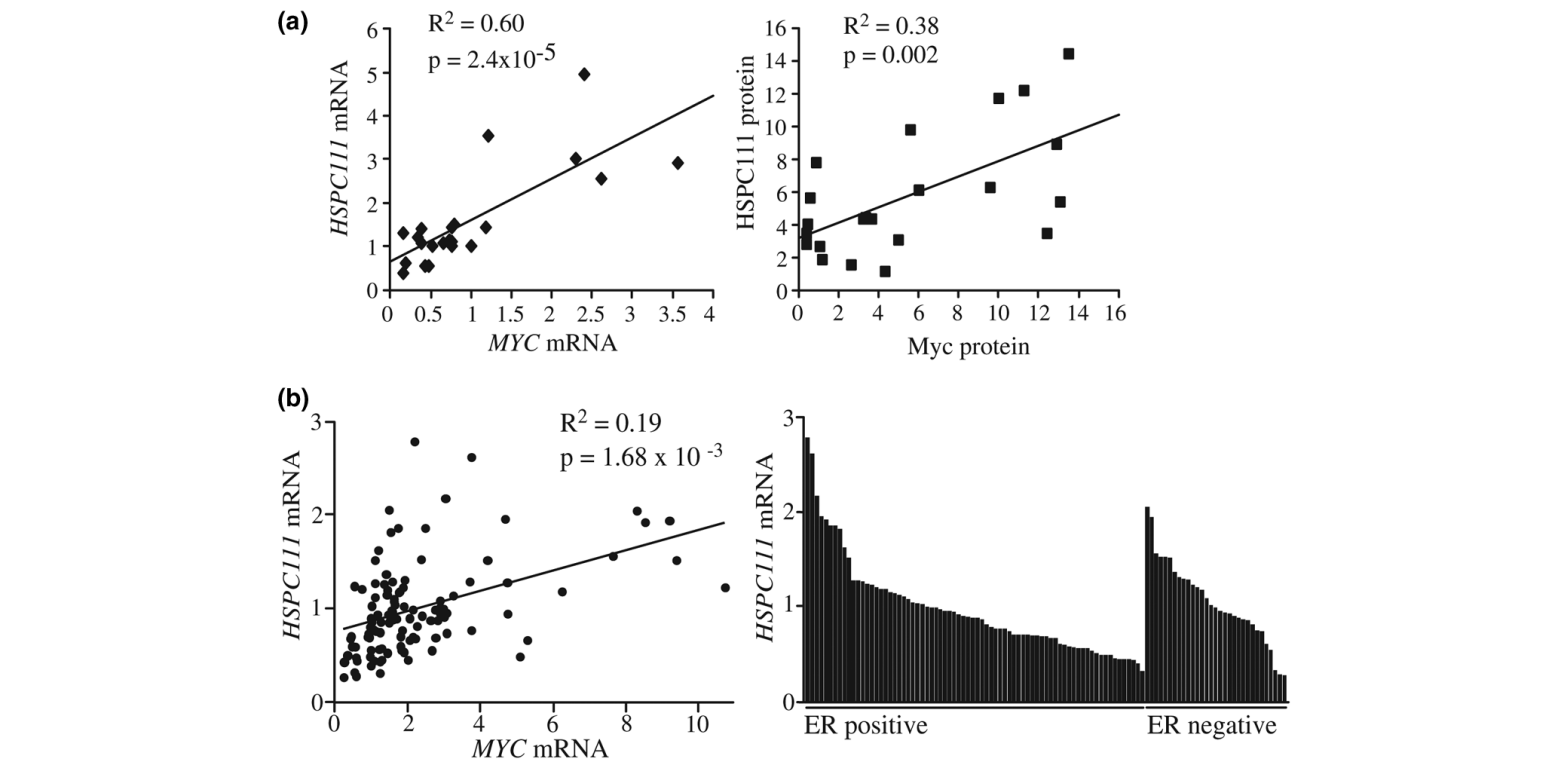
Correlation between HSPC111 and c-Myc expression in human breast cancer cell lines and tumor samples. **(a) **Expression of HSPC111 and Myc mRNA and protein was determined by quantitative real-time PCR and immunoblot, respectively, in a panel of 16 breast cancer cell lines with either estrogen receptor (ER)-negative or ER-positive status. **(b) **Correlation between *HSPC111 *and *MYC *mRNA expression in primary breast cancers, and distribution of *HSPC111 *mRNA expression levels amongst ER-negative and ER-positive cancers.

To further investigate *HSPC111 *as a Myc target gene in human breast cancer, we examined the expression of HSPC111 and Myc in a panel of breast cancer cell lines and primary breast cancers. A significant positive correlation between HSPC111 and Myc expression was observed at both the mRNA and protein level in 16 breast cancer cell lines (Figure 5a). We extended this study to examine the relationship between *HSPC111 *and *MYC *mRNA expression in a cohort of 105 primary breast carcinomas [19]. Comparative analysis of *HSPC111 *and *MYC *mRNA expression revealed a weak positive correlation in this breast cancer cohort (r^2 ^= 0.19, indicating that *MYC *contributed only about 20% of the variance in *HSPC111 *levels; Figure 5b). Furthermore, *HSPC111 *mRNA expression was distributed similarly across both ER-positive and ER-negative breast cancers (mean relative expression 0.94 ± 0.06 and 1.03 ± 0.08 respectively; Figure 5b).

We then utilized the cancer-profiling database Oncomine™ [31,32] and our own published studies [33,34] to further explore the relative expression of *HSPC111 *in normal versus cancer tissues from a range of published gene expression arrays. This confirmed that *HSPC111 *mRNA was significantly over-expressed in two other breast cancer cohorts [35,36] and in other steroid-regulated cancers, such as prostate [33,37] and ovarian carcinoma [34,38]. In addition, studies in other cancer types such as testis [39], liver [40], colon [41], and pancreas [42] revealed over-expression of *HSPC111*. Interestingly, some but not all of these studies showed a concurrent over-expression of *MYC *[37,39], suggesting that HSPC111 is regulated both by Myc-dependent and -independent pathways.

### HSPC111 expression is associated with poor survival in breast cancer patients

Datasets from two well characterized breast cancer cohorts were examined to assess whether the documented *HSPC111 *over-expression in breast cancer was associated with disease outcome. From the Uppsala cohort [21], two probesets that assessed expression of *HSPC111 *on the Affymetrix HGU133 microarrays satisfied quality control criteria for analysis. For both probesets, high expression of *HSPC111 *mRNA was associated with poor survival when modeled as a continuous variable using Cox proportional hazards analysis (probeset 203023_at: hazard ratio = 2.30, 95% CI 1.23 to 4.30; *P *= 0.0091; probeset 214011_s_at: hazard ratio = 3.13, 95% CI 1.62 to 6.06; *P *= 0.0007). None of the four probesets assessing *MYC *expression were associated with outcome when modeled as continuous variables (*P *> 0.13).

**Figure 6:**
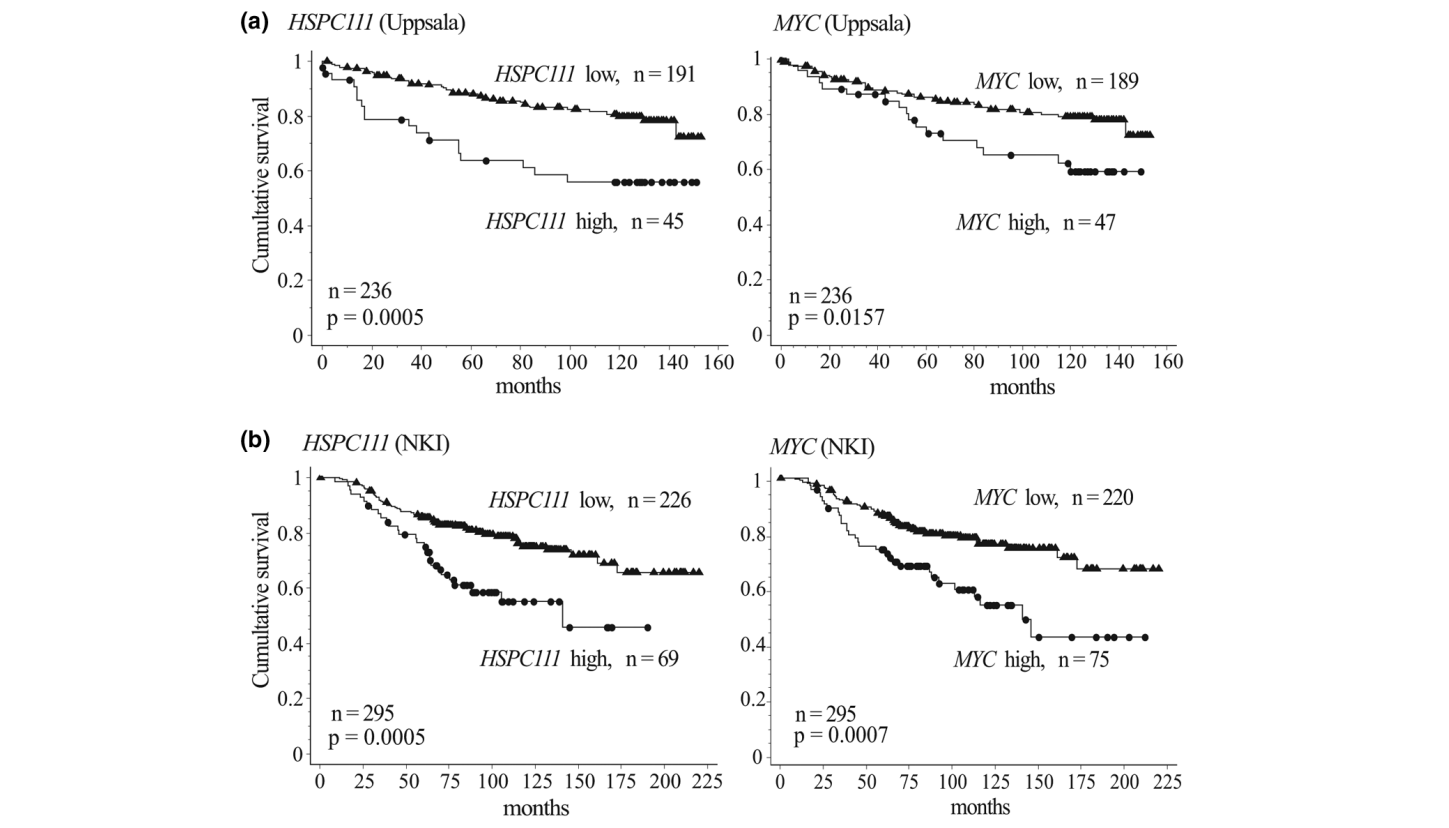
High HSPC111 expression is associated with poor survival in breast cancer cohorts. Kaplan-Meier survival curves of the relationship between *HSPC111 *and *MYC *mRNA expression and survival in two independent publicly available breast cancer cohorts: **(a) **Uppsala cohort and **(b) **the Nederlands Kanker Instituut (NKI) cohort.

To dichotomize data for both cohorts, a cutpoint was selected to identify approximately the upper quartile of *HSPC111 *expressors in both studies. As shown in Figure 6a, Kaplan-Meier analysis demonstrated that high expression of *HSPC111 *was associated with poor survival for both probesets (203023_at: 45/236 [19.1%] with high expression; *P *= 0.0005; 214011_s_at: 66/236 [28%]; *P *= 0.0137). Similarly, approximately the highest quartile was selected for *MYC*, reflecting the expected proportion of cancers that would probably have amplification of the gene. The only probeset that demonstrated an association between high *MYC *expression and a poor survival was 224340_at (47/236 [19.9%], *P *= 0.0157). Multivariate modeling identified that *HSPC111 *expression was an independent prognostic factor with the final resolved model shown in Table 1. *HSPC111 *was independent of the influence of high *MYC *expression on survival (shown as a bivariate model in Table 1).

**Table 1:** Multivariate analysis of clinicopathological parameters and HSPC111 expression in two breast cancer cohorts

Variable	Hazard ratio (95% confidence interval)	*P*
Uppsala cohort		

Tumor size > 20 mm	3.20 (1.65 to 6.21)	0.0006
Lymph node positive	3.13 (1.79 to 5.46)	< 0.0001
*HER2 *high expression	1.82 (1.02 to 3.27)	0.0444
		
*HSPC111 *high expression	2.29 (1.30 to 4.03)	0.0043
*HSPC111 *high expression	2.51 (1.43 to 4.41)	0.0014
*MYC *high expression	1.89 (1.06 to 3.36)	0.0305

NKI cohort		

Tumour size > 20 mm	1.90 (1.10 to 3.28)	0.0223
Tumour grade > 2	2.04 (1.22 to 3.44)	0.0070
ER positive	0.67 (0.38 to 1.18)	0.1636
PR positive	0.56 (0.32 to 1.00)	0.0490
*HER2 *high expression	1.46 (0.87 to 2.43)	0.1493
*HSPC111 *high expression	1.29 (0.79 to 2.12)	0.3074
		
*HSPC111 *high expression	1.89 (1.17 to 3.08)	0.0097
*MYC *high expression	1.83 (1.14 to 2.93)	0.0118

NKI, Nederlands Kanker Instituut.

In the dataset from The Nederlands Kanker Instituut cohort (NKI [22]), one probe assessed expression of *HSPC111*. High expression of *HSPC111 *mRNA was associated with poor survival when modeled as a continuous variable using Cox proportional hazard analysis (hazard ratio = 16.6, 95% CI 3.25 to 85.26; *P *= 0.0007). High *MYC *expression was also associated with poor survival (hazard ratio = 2.63, 95% CI 1.16 to 5.92; *P *= 0.02). Adopting a similar strategy for dichotomization of gene expression variables in this cohort demonstrated that, as with the Uppsala cohort, high *HSPC111 *expression (which occurred in 69/295 [23.4%] cases) was associated with poor survival (*P *= 0.0005), and high *MYC *expression (in 75/295 [25.4%] cases) was also associated with poor survival (*P *= 0.0007). Multivariate modeling demonstrated that in this cohort, *HSPC111 *expression was not an independent prognostic factor with the final resolved model shown in Table 1. However, as with the Uppsala cohort, *HSPC111 *was independent of the influence of high *MYC *expression on survival, which is shown as a bivariate model in Table 1.

## Discussion

Although it is now well established that the mitogenic effects of estrogen play a pivotal role in the initiation and progression of breast cancer, how these effects are mediated at the molecular level remains to be fully elucidated. The transcription factor c-Myc is a prominent player in the response of breast cancer cells to estrogen, mimicking the effects of estrogen on cell cycle progression [12] and conferring resistance to antiestrogens *in vitro *[14,15,43]. Thus, identification and characterization of key downstream effectors of estrogen and Myc action will not only provide a greater insight into estrogen effects on mitogenesis and survival, but could also lead to an enhanced understanding of the mechanisms governing endocrine resistance [8].

In a search for estrogen-target genes that are regulated secondarily to estrogen's induction of c-Myc, we identified a novel gene of unknown function, namely *HSPC111*, which was among the most highly regulated estrogen and Myc target genes in our model [12] (Musgrove EA, Sergio CM, Butt AJ, Sutherland RL; unpublished data). *HSPC111 *is rapidly (within 3 hours) upregulated in response to treatment with estrogen (about threefold) and induction of Myc (about fourfold). However, the response to estrogen is abrogated in the presence of Myc siRNA, providing strong evidence that estrogen stimulates HSPC111 expression via its well documented upregulation of Myc. This conclusion is further supported by our demonstration of functional E-boxes in the *HSPC111 *promoter, and Myc-responsive promoter activity, identifying *HSPC111 *as a direct transcriptional target of Myc. Although gene expression profiling has recently identified *HSPC111 *as a target of estrogen [44] and Myc [45], this is the first report demonstrating that estrogen's effects on HSPC111 are dependent upon a direct transcriptional activation by Myc.

Although HSPC111 is a previously uncharacterized protein, it is known to reside in the nucleolus [46]. In an attempt to elucidate a cellular role for HSPC111, we further investigated its subcellular localization. The nucleolus is the center of ribosomal biosynthesis and assembly [29]. HSPC111 did not colocalize with either NPM/B23 or fibrillarin, both of which are known to play a role in ribosomal biosynthesis. However, sucrose density fractionation demonstrated that HSPC111 is part of a RNA-dependent complex sedimenting in the 40 to 80S region, which also contains preribosomal ribonucleoprotein particles [29]. In addition to driving cell division, Myc plays a crucial role in controlling cell growth and protein synthesis [47]. Thus, the acute transcriptional regulation of HSPC111 by Myc may represent part of a coordinated stimulation of ribosome biogenesis [47], occurring concurrently with its stimulation of cell proliferation. However, whether HSPC111 has a role in the ribosomal biosynthesis pathway is not clear from these studies and requires further investigation.

Recent studies have emphasized an important link between nucleolar function, in particular ribosomal biogenesis, and cell cycle control, and several genes coordinately regulate both processes. For example, disruption of the nucleolar PeBoW complex, consisting of Pes1, Bop1 and WDR12, blocks both rRNA processing and cell cycle progression [48,49]. Given the proliferative role of Myc in our model and our data suggesting HSPC111 interacts with RNA in the nucleolus, we questioned whether HSPC111 might play a role in Myc's effects on cell cycle progression. However, modulation of HSPC111 expression had no effect on cell proliferation end-points. We detected no effect of constitutive HSPC111 expression on proliferation, and although it is possible that the level of over-expression achieved was not sufficient for a detectable increase in proliferation rate, HSPC111 expression was not required for cell cycle progression, and neither was its downregulation required for antiestrogen inhibition of proliferation. These data are supported by Schlosser and coworkers [45], who identified *HSPC111 *as a Myc target gene in the human B-cell line P493-6 under conditions in which Myc induces cell growth but not cell proliferation [50]. Furthermore, they suggested that, even if HSPC111 does play a role in rRNA synthesis, there is either an element of functional redundancy in its role or it is not rate-limiting for cell cycle progression. Indeed, although adequate cell growth is essential for proliferation, it is not sufficient, requiring additional proliferative signals for cell cycle progression to proceed [51]. These data emphasize the complexity of the Myc phenotype, even within the relative restrictions of our model system, and support the concept that the coordinated regulation of multiple effector genes is required to recapitulate Myc functions [52].

To address further a potential role of HSPC111 in cancer, we initially identified a strong positive correlation between *MYC *mRNA and both HSPC111 mRNA and protein in breast cancer cell lines, raising the possibility that HSPC111 expression might be a useful surrogate marker of Myc over-expression in breast cancer. However, the relationship at the mRNA level was less robust in primary breast cancer (r^2 ^= 0.19 versus 0.60 for primary cancer versus cell lines). An extension of this analysis to published datasets from a number of other cancers identified that elevated expression of HSPC111 was a feature of several cancers including those of the breast, prostate, ovary, testis, liver, colon, and pancreas [31,41], but this was not always associated with *MYC *over-expression. Thus, HSPC111 over-expression appears to be a common feature of many cancers but its relationship to aberrant Myc function, which is only contributed in part by *MYC *mRNA levels, remains to be elucidated. More importantly, HSPC111 over-expression was a strong predictor of an adverse outcome in two cohorts of breast cancer patients on univariate analysis and remained significant in a multivariate model in the Uppsala cohort. These effects were independent of *MYC *mRNA over-expression, which is in support of our conclusions from other cancers. Whether HSPC111 over-expression is functionally associated with disease progression remains an open question. The data presented here failed to support a role in cell proliferation or endocrine sensitivity, but other aspects of the biology of tumor progression require further investigation. It is well established that aberrant cell growth (increased/dysregulated ribosome biogenesis and protein synthesis) are common features of cancer. Because our preliminary data point to nucleolar localization of HSPC111 in association with ribonucleoproteins, it may be either functionally associated with these processes or a marker of aberrant cell growth regulation in general. In any event, further investigation of the normal physiological role of HSPC111 in nucleolar function and the functional consequences of overexpression on cellular growth control are warranted.

## Conclusion

In summary, we have identified *HSPC111 *as an estrogen-responsive, Myc target gene in breast cancer cells. Although the precise function of HSPC111 remains unclear from our studies, its over-expression is common in a number of cancer cell types, and its association with poor outcome in breast cancer cohorts warrants its further analysis as an effector of estrogen and Myc action in both normal and neoplastic growth.

## Abbreviations

bp = base pairs; CAD = carbamoyl phosphate synthetase-aspartate transcarbamylase-dihydroorotase; ChIP = chromatin immunoprecipitation; CI = confidence interval; ER = estrogen receptor; GAPDH = glyceraldehyde 3-phosphate dehydrogenase; NKI = Nederlands Kanker Instituut; NPM = nucleophosmin; PCR = polymerase chain reaction; si = small interfering.

## Competing interests

The authors declare that they have no competing interests.

## Authors' contributions

AJB, RLS, and EAM conceived the study, participated in its design, coordination and interpretation, and drafted the manuscript. AJB additionally generated cell lines over-expressing HSPC111. CMS performed analyses of HSPC111 expression, sucrose density gradient fractionation, and analyses of cells following HSPC111 over-expression or knock-down. CKI performed analyses of HSPC111 transcriptional regulation, participated in sucrose density fractionation, measured gene expression in breast cancers, and helped to draft the manuscript. LRA performed immunolocalization experiments, participated in HSPC111 knock-down experiments, and measured gene expression in breast cancer cell lines. CMM analyzed HSPC111 expression in breast cancers. AJR generated and characterized the HSPC111 antibody. MN and TP participated in the design and interpretation of sucrose density fractionation. AVB examined HSPC111 association with disease outcome and helped to draft the manuscript. All authors read and approved the final version.
